# Myogenesis gone awry: the role of developmental pathways in rhabdomyosarcoma

**DOI:** 10.3389/fcell.2024.1521523

**Published:** 2025-01-20

**Authors:** Annika L. Gustafson, Adam D. Durbin, Kristin B. Artinger, Heide L. Ford

**Affiliations:** ^1^ Department of Pharmacology, University of Colorado Anschutz Medical Campus, Aurora, CO, United States; ^2^ Molecular Biology Graduate Program, University of Colorado Anschutz Medical Campus, Aurora, CO, United States; ^3^ Medical Scientist Training Program, University of Colorado Anschutz Medical Campus, Aurora, CO, United States; ^4^ Division of Molecular Oncology, Department of Oncology, St. Jude Children’s Research Hospital, Memphis, TN, United States; ^5^ Department of Diagnostic and Biological Sciences, University of Minnesota School of Dentistry, Minneapolis, MN, United States

**Keywords:** rhabdomyosarcoma, myogenesis, developmental heterogeneity, transcription factor (TF), targeted therapeutic agents

## Abstract

Rhabdomyosarcoma is a soft-tissue sarcoma that occurs most frequently in pediatric patients and has poor survival rates in patients with recurrent or metastatic disease. There are two major sub-types of RMS: fusion-positive (FP-RMS) and fusion-negative (FN-RMS); with FP-RMS typically containing chromosomal translocations between the *PAX3/7-FOXO1* loci. Regardless of subtype, RMS resembles embryonic skeletal muscle as it expresses the myogenic regulatory factors (MRFs), MYOD1 and MYOG. During normal myogenesis, these developmental transcription factors (TFs) orchestrate the formation of terminally differentiated, striated, and multinucleated skeletal muscle. However, in RMS these TFs become dysregulated such that they enable the sustained properties of malignancy. In FP-RMS, the *PAX3/7-FOXO1* chromosomal translocation results in restructured chromatin, altering the binding of many MRFs and driving an oncogenic state. In FN-RMS, re-expression of MRFs, as well as other myogenic TFs, blocks terminal differentiation and holds cells in a proliferative, stem-cell-like state. In this review, we delve into the myogenic transcriptional networks that are dysregulated in and contribute to RMS progression. Advances in understanding the mechanisms through which myogenesis becomes stalled in RMS will lead to new tumor-specific therapies that target these aberrantly expressed developmental transcriptional pathways.

## 1 Introduction

### 1.1 Overview of rhabdomyosarcoma

Rhabdomyosarcoma (RMS) is the most common pediatric soft-tissue sarcoma, accounting for nearly half of all pediatric soft-tissue sarcoma cases (Amer et al., 2019; Kashi et al., 2015). Primary RMS occurs most commonly in the head and neck (∼30%), followed by the genitourinary region (∼25%), and then the extremities (∼20%) (Pappo et al., 1995; Sultan et al., 2009). Standard of care treatment for RMS patients remains the use of cytotoxic combination chemotherapy (vincristine, actinomycin-D, and cyclophosphamide), along with external-beam radiation and surgery (Miwa et al., 2020). The long-term effects of these therapies are severe - chemotherapy regimens may result in a host of short and long-term toxicities including infertility and secondary neoplasia, surgery may cause disfigurement, organ loss or dysfunction, and radiotherapy results in disfigurement, developmental disruptions and a risk of secondary malignancy (Saab et al., 2011). Despite these toxicities, current treatment regimens have resulted in a 5-year overall survival of 85% for children with localized disease. However, patients with recurrent or metastatic disease do not fare as well, with 5-year survival rates of 17% and 30% respectively (Pappo et al., 1999; Crist et al., 2001; Di Carlo et al., 2023; Bisogno et al., 2019). Critically, there are limited targeted therapies for the treatment of RMS, the development of which may not only lessen the long-term harm of using non-tumor specific therapies in pediatric patient populations but may also serve to improve the survival of patients with recurrent and metastatic disease.

A hallmark of RMS is its characteristic resemblance to a neoplastic version of skeletal muscle; however the histologic and molecular characteristics of tumors display variance (Parham, 2001). RMS express DESMIN, MYOD1, and MYOG, proteins associated with myogenesis, resulting in a striking similarity to developing skeletal muscle (Agaram, 2022). RMS is subclassified into four dominant distinct histologic subtypes, embryonal (eRMS), alveolar (aRMS), spindle cell/sclerosing (sp/scRMS), and pleomorphic (pRMS) (Agaram, 2022; Choi and Ro, 2021). The two most common subtypes of RMS, eRMS (found in ∼60% of patients) and aRMS (found in ∼30% of patients), differ in their molecular drivers (Ognjanovic et al., 2009). Alveolar RMS are driven, in 60% of cases, by an oncogenic chromosomal translocation between paired box 3 (*PAX3)* and the forkhead transcription factor (*FOXO1)* [*t(2;13) (q35;q14)*] or, in 20% of aRMS cases, by a translocation between paired box 7 (*PAX7*) and *FOXO1* [*t(1;13) (p36;q14)*] (Kashi et al., 2015). The remaining patients typically display more rare variant translocations incorporating other regulators, such as *NCOA1*, *NCOA2, INO80D* or others, with very few containing no detectable fusions (Saab et al., 2011; Agaram, 2022; Skapek et al., 2019). Beyond the hallmark *PAX3/7-FOXO1* chromosomal translocation, a third of alveolar RMS tumors harbor amplifications of *MYCN* (Driman et al., 1994; Williamson et al., 2005). Apart from the genomic alterations already discussed, aRMS exhibit very few somatic mutations, copy number variants, or structural variations (Chen et al., 2013). Histologically, aRMS are characterized on hematoxylin and eosin (H&E) stain by aggregates of small, round blue cells that occupy nests outlined by fibrous septa forming structures reminiscent of pulmonary alveoli – hence the name (Saab et al., 2011; Parham, 2001; Agaram, 2022).

In contrast, eRMS often harbor mutations in receptor tyrosine kinase and cytoplasmic signaling (*NF1, FGFR4, PIK3CA*) and transcriptional regulators (*TP53*, *CTNNB1*) (Chen et al., 2013; Shukla et al., 2012; Ignatius et al., 2018). While eRMS lack any recurrent oncogenic chromosomal translocations, these tumors have a greater number of somatic mutations as well as structural and copy number variations compared to aRMS (Chen et al., 2013; Shern et al., 2014). Metastatic eRMS commonly have mutations in *KRAS*, *NRAS*, or *HRAS* (Kashi et al., 2015; Shern et al., 2021). Histologically, eRMS is characterized by tumor cells that reproduce a broader range of myogenic stages compared to aRMS, with morphologically round and spindle shaped cells and scattered rhabdomyoblasts surrounded by myxoid stroma (Saab et al., 2011; Parham, 2001; Agaram, 2022). Importantly, some tumors that have been histologically identified as aRMS, but lack any chromosomal translocation, are much more similar in clinical presentation, outcome, and gene expression to eRMS than to other fusion-driven aRMS (Williamson et al., 2010). Therefore, current therapeutic strategies have used the presence or absence of chromosomal fusions to define subtypes of RMS – either fusion positive (FP)-RMS, which are largely aRMS, or fusion negative (FN)-RMS, which are largely eRMS.

**FIGURE 1:**
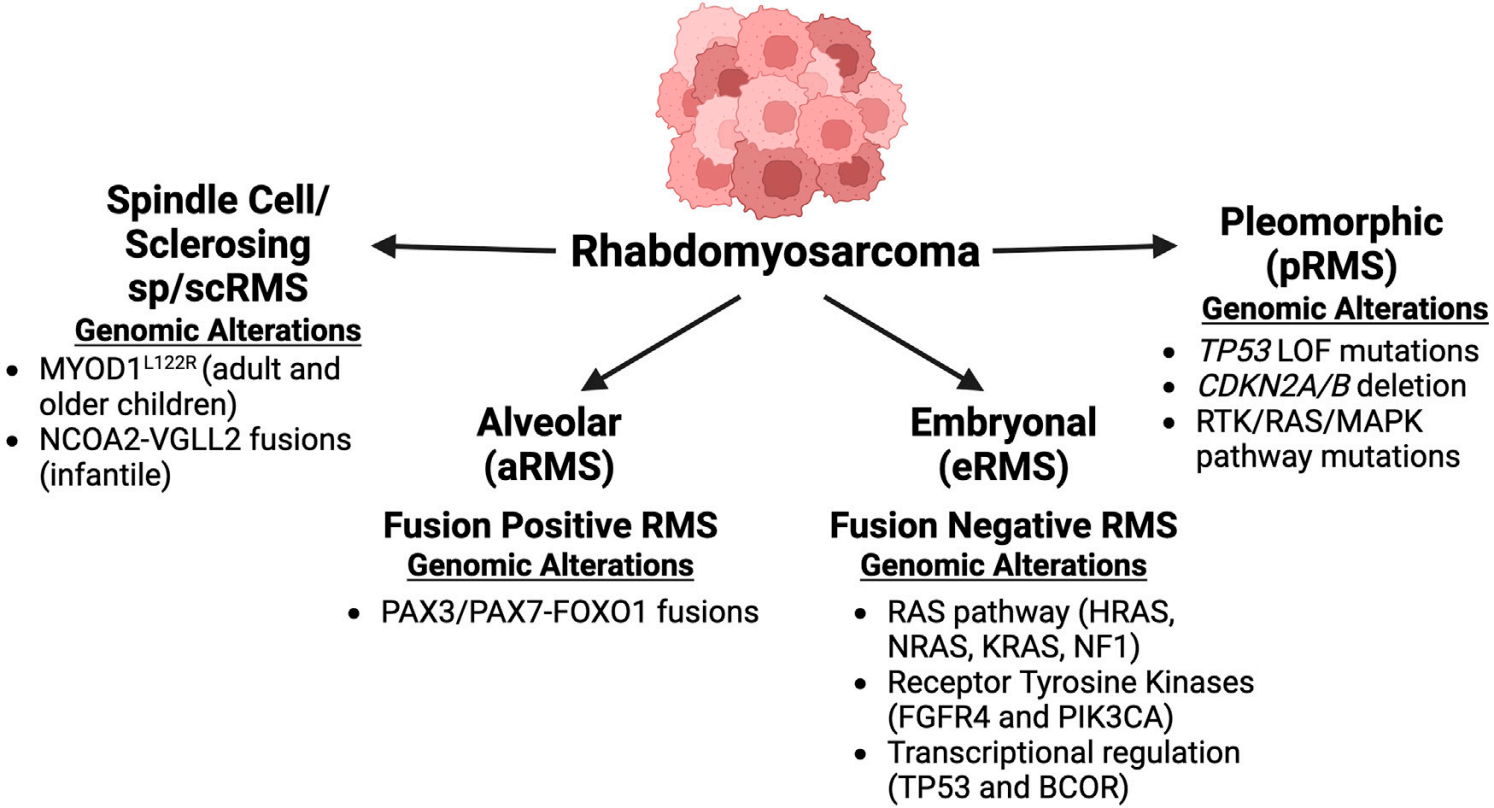
Rhabdomyosarcoma subtypes with prominent genomic alterations: Rhabdomyosarcoma can be split into four different subtypes, alveolar, embryonal, pleomorphic, and spindle cell/sclerosing. Each subtype has different genetic alterations, prognosis and occurs in different patient populations. The two predominant subtypes are alveolar and embryonal, which are molecularly subcategorized as fusion-positive (alveolar) and fusion-negative (embryonal). Figure created with Biorender.com.

Other RMS subtypes do not fit neatly into the FN-RMS or FP-RMS designations. Instead, sp/scRMS and pRMS are notable for mutations in myogenic pathways and clinical presentations which differ strikingly from FN-RMS and FP-RMS. Spindle cell/sclerosing RMS (sp/scRMS) is notable for a recurrent *MYOD1^L122R^* mutation which is associated with exceptionally poor prognosis, or *NCOA2* fusions (*VGLL2-NCOA2, TEAD1-NCOA2,* and *SRF-NCOA2*). *NCOA2* fusions occur in infantile spRMS and are associated with a good prognosis (Di Carlo et al., 2023; Shukla et al., 2012; Shern et al., 2014; Alaggio et al., 2016). The *MYOD1^L122R^* mutation alters the DNA-binding basic domain of MYOD1 to drive ectopic activity that is hypothesized to resemble that of MYC proteins, another family of basic-helix-loop-helix (bHLH) TFs (Kohsaka et al., 2014). In contrast to the primarily pediatric subtypes of FN-, FP- and sp/scRMS, pRMS almost exclusively occurs in adult patients, arising in the deep tissues of the extremities. This tumor is typically associated with activating mutations in *KRAS*, and is associated with clinically unfavorable outcomes (Sultan et al., 2009; Egas-Bejar and Huh, 2014). Thus, RMS subtypes differ in their histology, presentation, and clinical outcomes reflecting the different biology driving the disease and potentially also various cells of origin (Figure 1). Understanding the mechanism through which diverse RMS subtypes are held in an undifferentiated state is a promising route through which new therapeutic targets may be identified.

### 1.2 Developmental paradigms in rhabdomyosarcoma

RMS resembles embryonic skeletal muscle molecularly and histologically (Stewart et al., 2018). However, primary RMS can occur in regions of the body where skeletal muscle is absent; reported primary tumor sites include the orbit of the eye, salivary gland, bladder, testis, or prostate (Amer et al., 2019; Miwa et al., 2020; Skapek et al., 2019). The histology of RMS and the locations in which primary tumors arise have made it challenging to clearly identify a cell of origin. Some studies suggest that the cell of origin is a dedifferentiated myocyte or a myogenic progenitor that constitutively activates classic cancer pathways allowing the tumor cell to evade death signals and proliferate indefinitely (Ignatius et al., 2018; Tenente et al., 2017; Ignatius et al., 2012; Keller et al., 2004a; Keller et al., 2004b). Other studies suggest that aberrant activation of a myogenic program can also occur in mesenchymal stem cells, or endothelial progenitors, resulting in RMS formation (Saab et al., 2011; Drummond et al., 2018; Charytonowicz et al., 2009; Hatley et al., 2012). These data suggest that the cell of origin may be distinct in different anatomic sites, resulting in a spectrum of mutations consistent with different subtypes of tumors. To this end, FP-RMS typically arises in the extremities, while FN-RMS is more common in the genitourinary systems and head and neck (Agaram, 2022). This observation lends support to the hypothesis that tumors arising in different primary sites may have distinct cells of origin, with stereotyped mutations occurring in susceptible cells of origin at a specific developmental timepoint at which the cell displays oncogenic competence. The common endpoint, despite these complicating factors, is that RMS clearly asserts a myogenic lineage identity.

Single-cell RNA sequencing (scRNAseq) experiments have provided novel insights into the intratumoral malignant heterogeneity of RMS. Three recent independent studies performed scRNAseq on RMS patient-derived xenografts, patient samples, and RMS cell lines, to identify a progenitor-like population, a differentiated population, and a population of proliferating cells (Wei et al., 2022; Danielli et al., 2023; Patel et al., 2022). Two of these studies also identified a population of non-proliferating cells that lack a known transcriptional signature, termed “Ground-State” (Wei et al., 2022; Danielli et al., 2023). A meta-analysis of these three datasets identified five distinct subpopulations in RMS including progenitor*,* proliferative, differentiated, apoptotic, and a ground subpopulation, the latter of which doesn’t enrich for any known signature (Danielli et al., 2024). Within these five subpopulations there are “transitory” progenitor and differentiated subpopulations, emphasizing that there is a partially sustained myogenic process in these tumors (Danielli et al., 2023; Danielli et al., 2024). This finding is further emphasized by *in silico* RNA-velocity analysis performed on FN-RMS revealing a conserved myogenic program resulting in myogenic progenitor-like cells that unidirectionally differentiate into myoblast- and myocyte-like tumor cells (Patel et al., 2022).

The authors of the meta-analysis study further created a “muscle lineage score” by calculating the difference between differentiated and progenitor signature scores for every single-cell profile in the dataset. Importantly, this analysis demonstrated that FP-RMS samples had a more differentiated muscle-lineage score than FN-RMS when compared as either single-cell or pseudo-bulk data sets (Danielli et al., 2024). To further dissect the myogenic states present in RMS, the authors compared the scRNAseq datasets to an annotated normal human myogenic development scRNAseq dataset and found that in FN-RMS, populations of cells that resemble skeletal mesenchymal cells, myogenic progenitors, myoblasts, and myocytes exist (Danielli et al., 2024). In contrast, FP-RMS almost entirely lack any cells that resemble skeletal mesenchymal cells, but these tumors do have populations of cells that resemble more differentiated myoblasts and myocytes (Danielli et al., 2024). These data match previous studies where RMS scRNAseq data was compared to a mouse organogenesis scRNAseq atlas, and demonstrated that FN-RMS exhibit a broader range of myogenic stages, than the narrower and later stages present in FP-RMS (Patel et al., 2022). Interestingly, there is also a unique population of cells within FP-RMS that adopt a more neuronal-like identity (Wei et al., 2022). The significance of a neuronal-like population in FP-RMS is not yet clear, though this subpopulation displays the highest signature score for fusion-oncogene activity, suggesting a potentially important, yet currently unknown, role of PAX3/7-FOXO1 (Danielli et al., 2024). Understanding the developmental hierarchies present in RMS provides insight into oncogenesis and has the potential to inform targeted therapy selection and development.

The sustained progenitor-like subpopulation of cells in RMS – particularly FN-RMS – contributes to disease recurrence and resistance to chemotherapy. A tumor propagating cell (TPC), defined as a tumor cell capable of self-renewal, proliferation, and that can produce all heterogenous tumor cell types within a tumor, was identified in FN-RMS as the less differentiated, skeletal muscle mesenchymal stem cell-like tumor cell (Wei et al., 2022). In FN-RMS, these less differentiated tumor cells express *EGFR* and are selectively resistant to chemotherapy regimens (Patel et al., 2022). Treatment of orthotopic patient-derived xenografts with a combination of standard of care chemotherapy regimens and an EGFR inhibitor resulted in significantly improved survival (Patel et al., 2022). These studies provide compelling evidence that targeting the TPC population is key to preventing recurrence and metastasis in RMS and that understanding the role of development in tumorigenesis may be key in the development of targeted therapeutics. To date, a TPC has not yet been identified in FP-RMS, but its identification may be highly informative to why FP-RMS patients have significantly worsened outcomes compared to FN-RMS patients. The degree to which developmental processes are conserved varies between RMS tumor subtypes and individual tumors, implying that there is variation in how myogenic development is perturbed across RMS.

### 1.3 Embryonic myogenesis

Formation of terminally differentiated contractile skeletal muscle is a highly regulated stepwise process that requires cell-intrinsic and cell-extrinsic signals. Muscle specification begins in the paraxial mesoderm, which is a transient bilateral domain that flanks the neural tube. The paraxial mesoderm undergoes cyclic segmentation into somites by a “segmentation clock,” which generates pulses of NOTCH, FGF, and WNT (Chal and Pourquié, 2017; Dequéant et al., 2006). The formation of somites occurs rostral to caudal, and is followed by further somitic segmentation into the sclerotome – the structure that forms the axial skeleton and tendons – and the dermomyotome – which gives rise to the dermis of the back, brown fat, and skeletal muscle (23). Somitic segmentation into the sclerotome and dermomyotome is induced by WNT, Bone morphogenic protein (BMP), and Sonic Hedgehog (SHH) signaling to the somite from surrounding structures (Chal and Pourquié, 2017; Sadler, 2015; Chiang et al., 1996). WNT signaling from the neural tube and ectoderm maintains the dermomyotome fate (Hirsinger et al., 2000). SHH signaling represses dermomyotome identity and promotes specification of the sclerotome identity (Fan and Tessier-Lavigne, 1994; Johnson et al., 1994; Murtaugh et al., 1999). Following formation of the dermomyotome, primary myogenesis, a process where the myotomes and limb muscles form, occurs (Chal and Pourquié, 2017). During primary myogenesis, *PAX3* expressing cells from the dermomyotome migrate ventrally to form the myotome or into the limb buds to form limb muscles (Chal and Pourquié, 2017; Horst et al., 2005; Hutcheson et al., 2009). Following primary myogenesis, secondary myogenesis occurs where skeletal muscle further develops on the scaffold established during primary myogenesis. During secondary myogenesis, myogenic progenitors decrease expression of *PAX3* and increase *PAX7* expression (Kelly et al., 1997; Messina et al., 2010). These *PAX7+* myogenic precursors fuse to each other or to existing primary myofibers to form secondary myofibers (Van Horn and Crow, 1989). The remaining *PAX7+* cells will go on to form a pool of adult skeletal muscle stem cells, termed satellite cells (Relaix et al., 2005).

In zebrafish, a model organism commonly used to study both myogenesis and RMS, the process of muscle development is similar to that of amniotes. Zebrafish myogenesis is initiated in the paraxial mesoderm, which undergoes segmentation into somites, which then give rise to the myotome where primary myogenesis occurs (Keenan and Currie, 2019). One important distinction between mammalian skeletal muscle and zebrafish skeletal muscle is that in mammals, fast and slow-twitch skeletal muscle fibers intermix in muscle bundles, whereas in zebrafish fast-twitch and slow-twitch muscle fibers are spatially segregated (Keenan and Currie, 2019). In zebrafish, slow-twitch skeletal muscle development is initiated by Hedgehog (Hh) signaling from the notochord, which initiates expression of *prdm1a* and commits the muscle precursors to a slow-twitch skeletal muscle fate (Keenan and Currie, 2019). In contrast, retinoic acid (RA) produced by the paraxial mesoderm induces Fgf8 signaling, resulting in cells taking on a fast-twitch muscle fate (Keenan and Currie, 2019). Essential to secondary myogenesis in zebrafish is the external cell layer (ECL), which is roughly the equivalent of the dermomyotome (Keenan and Currie, 2019). Zebrafish may undergo secondary myogenesis throughout their lives resulting in dramatically increased body size, and importantly, stem cell populations that contribute to life-long secondary myogenesis comprise the ECL and *Pax7+* satellite cells that are dispersed throughout the myotome (Keenan and Currie, 2019). Zebrafish are an invaluable tool for understanding the genetics underlying myogenesis and RMS and, despite differences in the spatial regulation of myogenesis, the temporal expression of myogenic TFs is highly conserved between mammals and zebrafish.

**FIGURE 2:**
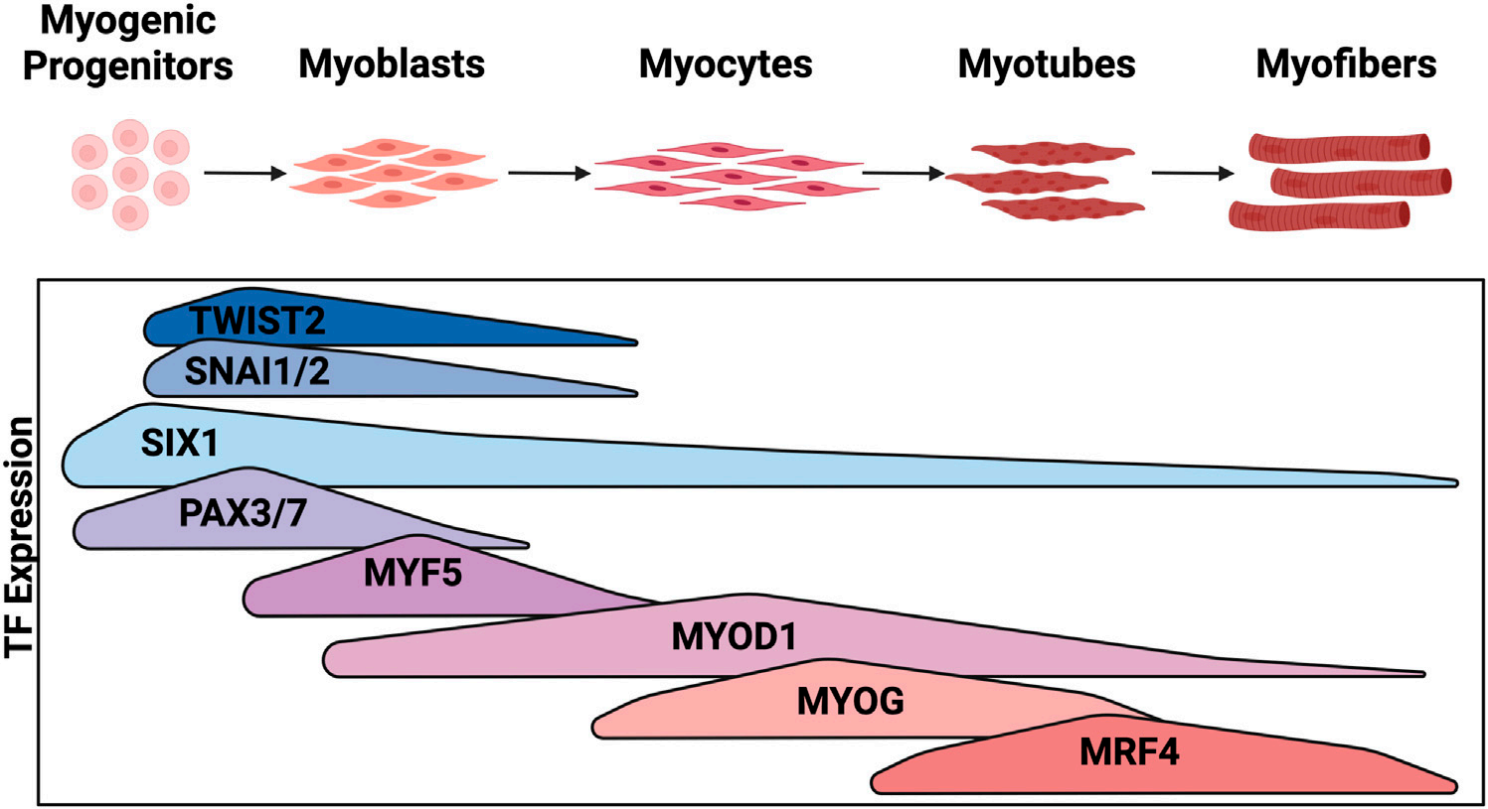
Expression of transcription factors during embryonic myogenesis: Embryonic myogenesis is regulated through the stepwise expression of myogenic TFs. The stage of myogenesis that each TF is expressed is depicted here. Figure created with Biorender.com.

Myogenic signaling pathways involve the precise temporal expression of developmental TFs (Figure 2). Myogenic cell specification begins in the somite with expression of *PAX3* and *SIX1*, homeobox TFs, which initiate expression of myogenic regulatory factors (MRFs) (Maroto et al., 1997; Grifone et al., 2005). MRFs are composed of a family of four basic-helix-loop-helix (bHLH) transcription factors (MYF5, MYOD1, MYOG, and MRF4) that initiate and execute myogenic lineage specification. *MYF5* expression is regulated by WNT, SHH, and BMP signaling and is the first of the MRFs to be expressed followed closely by *MYOD1* (Chiang et al., 1996; McDermott et al., 2005; Borycki et al., 1999; Krüger et al., 2001). MYF5 and MYOD1 both function to initiate expression of myogenic gene programs, however MYOD1 is a much more potent initiator of transcription (Maroto et al., 1997; Conerly et al., 2016; Buckingham and Rigby, 2014). MYOD1 is such a potent initiator of myogenesis that it can activate a skeletal muscle program in mouse embryonic fibroblasts (Davis et al., 1987). Early in muscle differentiation, MYOD1 is blocked from binding GC rich E-boxes that are enriched at differentiation genes by SNAI1 and SNAI2, resulting in MYOD1 maintenance at kinetically less favored AT rich E-boxes enriched at growth and proliferation genes (Soleimani et al., 2012). Thus, MYOD1 regulates vastly different gene sets depending on the stage of differentiation and which other TFs are present in the nucleus. MYOG is downstream of both MYOD1 and MYF5 and is required to develop mature muscle as it activates terminal differentiation gene targets. Finally, *MRF4* expression is regulated by MYOG and contributes to myocyte maturation while also negatively regulating *MYOG* expression (Zhang et al., 1995). Regulated expression of MRFs as well as their upstream activators *PAX3* and *SIX1* is critical for maintaining a progenitor population of cells, expanding early myogenic populations, and terminally differentiating cells into functional contractile skeletal muscle (Saab et al., 2011; Chal and Pourquié, 2017; Yu et al., 2006) (Figure 2).

Following the formation of terminally differentiated striated skeletal muscle, maintenance of skeletal muscle mass and recovery from injury is, in part, regulated by YAP/TAZ, effector coactivators of the Hippo signaling pathway (Kaya-Çopur et al., 2021; Koontz et al., 2013; Sun et al., 2017). YAP/TAZ, when dephosphorylated, enter the nucleus to interact with TEAD1-4 TFs (Sun et al., 2017) and regulate genes associated with proliferation and cellular differentiation. Both YAP and TAZ, in satellite cells, promote proliferation. However, in later stages of myogenesis TAZ promotes myogenic differentiation while YAP inhibits it (Sun et al., 2017). Each stage of myogenesis represents a potential point of dysregulation in RMS, and identifying conserved patterns of dysregulation across RMS tumors could inform the development of novel therapies.

## 2 Developmental transcription factors implicated in RMS

### 2.1 PAX3 and PAX7

PAX3 and PAX7 are members of the paired box family of TFs and are critical for initiation of muscle development in the dermomyotome and satellite cells. Data from FN-RMS suggest that PAX7 may be important for dictating the baseline transcriptional state. *PAX7* expression is increased in FN-RMS tumors compared to skeletal muscle, and is a genetic dependency in some FN-RMS tumors based on data from the Broad Institute’s Cancer Dependency Map (DepMap) (Langdon et al., 2021). Furthermore, In FN-RMS PAX7 has been hypothesized to be a core-regulatory TF, a member of a network of master TFs - termed a core-regulatory circuit (CRC) - that autoregulate themselves and each other, and serve to establish the majority of gene expression in the cell (Stewart et al., 2018; Gryder et al., 2019a). In FN-RMS, expression of *Pax7* is necessary for maintenance of the skeletal muscle identity as knock-out (KO) of *Pax7* in FN-RMS mouse models results in tumors that display smooth-muscle morphology, consistent with leiomyosarcoma (Langdon et al., 2021). When *PAX7* was knocked-down in human FN-RMS cell lines, proliferation was inhibited *in vitro* and *in vivo*, demonstrating the role of PAX7 as a dependency of FN-RMS.

The chromosomal translocation between *PAX3* and *FOXO1* [*t(2;13) (q35;q14)*] or *PAX7* and *FOXO1* [*t(1;13) (p36;q14)*] is key to sarcomagenesis in FP-RMS (Pappo et al., 1995). The chromosomal translocation driving FP-RMS fuses in-frame the NH2-terminal paired-box and homeodomain DNA-binding domains of PAX3 or, less commonly, PAX7, with the COOH-terminal transactivation domain of FOXO1 (Galili et al., 1993; Davis et al., 1994). The resultant fusion protein is a more potent transcriptional activator than wild-type (WT) PAX3 or PAX7 (Fredericks et al., 1995). One genetically engineered mouse model of FP-RMS consists of conditional *Pax3-Foxo1* expressed in more differentiated *Mrf4+* skeletal muscle cells (Keller et al., 2004b). When only *Pax3-Foxo1* is expressed in *Mrf4+* cells, tumors form at a low frequency. However, tumor formation frequency is increased through *Ink4a/ARF* mutations or *Trp53* loss of function mutations (Keller et al., 2004b). Limiting the generalizability of this model to human disease is the fact that in FP-RMS the most common genetic lesion, after *PAX3/7-FOXO1*, is a genetic amplification of *MYCN* or *CDK4*, or a loss of heterozygosity (LOH) at Chr11p15.5, a region that contains the known oncogene *IGF2* (Shern et al., 2014). Interestingly, expression of *Pax3-Foxo1* in *Pax7+* satellite cells did not result in FP-RMS formation, but did reduce the *Pax7+* satellite stem cell pool leading to animals with growth defects (Keller et al., 2004a). These data argue against satellite cells as the cell of origin for FP-RMS and indicate that the *Pax3-Foxo1* chromosomal translocation results in a novel oncogenic TF that perturbs normal myogenic differentiation.

Recent analysis shows that PAX3-FOXO1 can induce a myogenic-like identity in non-myogenic cells. A genetically engineered mouse model (GEMM) expressing an inducible *Pax3-Foxo1* in *aP2*+ (adipose-protein 2) endothelial progenitor cells demonstrates that following *Pax3-Foxo1* translocation, *aP2*+ cells are reprogrammed into functional *Pax7*+ myogenic stem cells, though robust FP-RMS formation was not observed (Searcy et al., 2023). Additionally, expression of *Pax3-Foxo1* in chick embryonic neural cells transdifferentiates the previously neural specified cells to a myogenic-like FP-RMS identity (Curto et al., 2020). Importantly, the ability of PAX3-FOXO1 to transdifferentiate cells to a myogenic cell fate has been shown to be unique to the fusion protein, as transdifferentiation does not occur with WT *Pax3* expression in chick embryonic neural cells (Curto et al., 2020). However, the PAX3-FOXO1 fusion protein is not sufficient for tumor formation, as PAX3-FOXO1 induction in chick embryonic neural cells and human fibroblasts causes cell-cycle inhibition, preventing cells from becoming malignant (Curto et al., 2020). Interestingly, expression of inducible *Pax3-Foxo1* in *Tek*+ cells (another marker for endothelial cells) along with *Cdkn2a* loss did not result in formation of functional *Pax7+* myogenic stem cells, but did result in robust FP-RMS formation, specifically in the snout (Searcy et al., 2023). Furthermore, cell cycle inhibition induced by PAX3-FOXO1 expression may be overcome with the addition of overexpressed *MYCN*, which is often amplified in RMS, or with Cyclin D1, *CCND1* (Curto et al., 2020). The induction of PAX3-FOXO1 expression in endothelial progenitors and spinal cord progenitors is particularly relevant to the modeling of FP-RMS as these tumors often arise in regions of the body that totally lack skeletal muscle, and single cell analysis of FP-RMS tumors reveals a subset of cells have a neural-like identity (Searcy et al., 2023; Curto et al., 2020).

Recent studies demonstrate that the PAX3-FOXO1 fusion protein enforces a FP-RMS myogenic-like cell fate in part by restructuring the epigenetic landscape. One aspect of genome organization is the folding of the genome into topologically associated domains (TADs), or chromatin neighborhoods where cis-regulatory regions interact (Rajderkar et al., 2023). In cells that lack the PAX3-FOXO1 onco-fusion protein, PAX3 and FOXO1 inhabit separate TADs, and their interactions are restricted to their respective genomic neighborhoods (Vicente-García et al., 2017). However, when the two proteins are fused, novel cis-regulatory element interactions occur whereby the *PAX3* promoter interacts with *FOXO1* regulatory regions, resulting in the restructuring of chromatin to form a novel TAD. The chromosomal translocation ultimately results in non-myogenic cells converting to a more myogenic state (Vicente-García et al., 2017). These data suggest that PAX3-FOXO1 may act as an oncogenic pioneer factor, a hypothesis further supported by data showing that PAX3-FOXO1 localizes to inactive chromatin and is capable of recognizing its motif on condensed chromatin, key characteristics of pioneer factors (Sunkel et al., 2021). To alter acetylation of histones and the epigenetic landscape, PAX3-FOXO1 recruits CBP/p300 and RNA Polymerase II, resulting in PAX3-FOXO1 target gene expression (Asante et al., 2023). The chromosomal translocation between *PAX3/7* and *FOXO1* combines a key developmental TF with a TF containing a potent transactivation domain, resulting in an altered chromatin landscape that enforces a FP-RMS identity reminiscent of developing skeletal muscle.

### 2.2 SIX1

SIX1 is a homeobox TF that transcriptionally regulates *PAX3*, *MYOD1*, *MYOG*, and *MRF4* in myogenesis and is overexpressed in both FP and FN-RMS (Grifone et al., 2005; Yu et al., 2006; Hsu et al., 2022; Liu et al., 2013; Ehinger et al., 2023; Relaix et al., 2013; Liu et al., 2010; Le Grand et al., 2012). Importantly, knock-down (KD) of *SIX1* in muscle progenitors decreases MRF expression and abrogates muscle differentiation (Liu et al., 2010; Le Grand et al., 2012). Analysis of the Broad Institute’s exome-wide CRISPR-Cas9 KO screen dataset demonstrates that RMS has an increased *SIX1* gene dependency (Hsu et al., 2022). SIX1 has largely been studied in the context of FN-RMS where it was found that KD of *SIX1* results in large-scale, genome-wide changes in transcription and leads to marked tumor cell differentiation (Hsu et al., 2022). Thus, loss of *SIX1*, in both zebrafish and mouse xenograft models, results in profound inhibition of tumor growth (Hsu et al., 2022). Mechanistically, SIX1 maintains a proliferative stem-like state in FN-RMS cells by maintaining MYOD1 at enhancers associated with stemness and proliferation and by preventing MYOD1 from binding cooperatively with MYOG at the promoters of differentiation genes (Hsu et al., 2022). Thus, SIX1 partially reproduces a normal myogenic process in RMS, where it is known to bind cooperatively with MYOD1 to activate growth and proliferation genes (Liu et al., 2013; Liu et al., 2010). However, in models of normal myogenesis, when *SIX1* is KD or KO, cells are locked in a non-proliferative stem-like state, as opposed to a non-proliferative terminally differentiated state as observed with *SIX1* KD in FN-RMS (Le Grand et al., 2012). These results indicate an important role for SIX1 in positively regulating proliferation, and suggest that either its levels, or its interaction with other context-specific TFs, guide whether its loss suppresses or enhances differentiation.

The cell cycle role of SIX1 has been documented in numerous contexts. SIX1 has been shown to repress p16 in mouse embryonic fibroblasts overexpressing HRAS and SIX1 (De Lope et al., 2019), thereby suppressing cellular senescence. In addition, SIX1 increases the expression of a number of cell cycle regulatory proteins including Cyclin D1, Cyclin A1, and c-Myc, and decreases the expression of inhibitors of the cell cycle such as p53 (Yu et al., 2006; Ford et al., 2000; Ford et al., 1998; Towers et al., 2015). The observed effects of SIX1 on key cell cycle genes is likely a conserved developmental function that is co-opted by tumors to maintain growth and proliferation.

In addition to regulating the proliferative and differentiation state of FN-RMS, SIX1 is known to contribute to the metastatic potential of this disease. SIX1 directly regulates *EZRIN*, a cytoskeletal organizer shown to be necessary for metastasis in a hepatocyte growth factor/scatter factor (*HGF/SF*)-transgenic, *Ink4a/Arf* deficient mouse model of FN-RMS (Yu et al., 2006; Yu et al., 2004). Adding relevance to the role of SIX1 in human disease, analysis of RNA-sequencing data from human RMS before and after relapse shows a statistically significant correlation between a *SIX1* KD transcriptional signature and decreased relapse (Hsu et al., 2022). Interestingly, SIX1 is highly expressed and a dependency in both FN-RMS and FP-RMS (Hsu et al., 2022). In support of a role for *SIX1* in FP-RMS, it was identified as a target of the PAX3-FOXO1 fusion protein (89). However, whether and how SIX1 contributes to FP-RMS progression has not been explored. In sum, SIX1 is a developmental TF that contributes to FN-RMS pathogenesis by rewiring the binding of MRFs, facilitating the evasion of cell-cycle arrest, and promoting the expression of pro-metastatic genes. It remains to be determined whether the functions of SIX1 in FN-RMS overlap with those in FP-RMS.

### 2.3 Myogenic regulatory factors: MYF5, MYOD1, and MYOG

In muscle development *MYF5* is the first of the MRFs to be expressed (Figure 2), and it is also expressed in a subset of RMS (Tenente et al., 2017; Maroto et al., 1997; Zibat et al., 2010). In transgenic zebrafish models of FN-RMS driven by *rag2-kRASG12D*, *myf5-GFP+* tumor cells were shown to have a greater tumor-propagating potential when compared to more differentiated myosin light chain 2 *(mylz2)-mCherry+* or intermediately differentiated *myf5-GFP+/mylz2-mCherry+* cells (Ignatius et al., 2012). Transgenic expression of *mylpfa:myf5*, a transgene that drives *myf5* expression in terminally differentiated, myosin light chain 11 (*mylpfa*) expressing cells, resulted in higher penetrance of *rag2-kRAS* ^*G12D*^ tumor formation (Tenente et al., 2017). Interestingly, the tumors that arose in *rag2-kRAS* ^*G12D*^ *;mylpfa-myf5* transgenic zebrafish were larger and exhibited a more differentiated morphology when compared to zebrafish only expressing transgenic *rag2-kRAS* ^*G12D*^, likely due to the forced expression of *myf5* in more differentiated *mylpfa* expressing cells (Tenente et al., 2017). The authors then showed by western blot analysis that in human RMS cell lines, protein expression of MYF5 and MYOD1 are mutually exclusive, suggesting that MYF5 may function similarly to MYOD1 (Tenente et al., 2017). Of note, this analysis included both FN-RMS and FP-RMS cell lines, and MYF5 protein expression was highest in Rh18, a reported aRMS cell line with fusion-negative status, that lacks MYOD1 expression (Tenente et al., 2017). Supporting the notion that MYF5 and MYOD1 are redundant in RMS, MYF5 in Rh18 cells and MYOD1 in RD cells are bound at similar promoter and enhancer regions genome wide, but most notably at enhancers associated with *MYOG* and *CCND2*, a CDK4/6-associated cyclin (Tenente et al., 2017). Redundancy between MYOD1 and MYF5 reproduces the developmental role of the two TFs whereby MYOD1 and MYF5 bind at similar locations, but exhibit differences in transactivation capacity (Conerly et al., 2016). A study comparing the transactivation activity of MYOD1 and MYF5 in the context of RMS would be potentially prognostic for patients whose tumors express one of these two TFs and would be of therapeutic relevance should a drug targeting MYOD1 in RMS be developed.

Most RMS tumors are dependent on MYOD1 for growth and proliferation. *MYOD1* is the most prominent gene dependency in RMS cells, in the DepMap exome-wide CRISPR-Cas9 KO screen dataset (Hsu et al., 2022; Dharia et al., 2021). MYOD1 is overexpressed in both major RMS subtypes and cells are dependent on MYOD1 for cell cycle progression and survival. Following KD of *MYOD1* in FN-RMS, cells exhibited decreased proliferation, cell-cycle arrest, decreased tumor sphere formation, and increased cell death (Tenente et al., 2017). In FN-RMS, MYOD1 drives proliferation and cell-survival, and is prevented from activating its later myogenic differentiation targets (Tenente et al., 2017; MacQuarrie et al., 2013). How MYOD1 functions in RMS cells to maintain cellular proliferation is a key question in the field of RMS biology.

MYOD1 is a bHLH TF, that forms a heterodimer with E-proteins to bind the E-box motif and activate muscle-specific genes (Shklover et al., 2007). In FN-RMS cell lines, when there are limited available E-proteins with which MYOD1 can form a heterodimer, MYOD1 is inhibited from binding DNA and activating downstream myogenic transcriptional targets (Tapscott et al., 1993; Yang et al., 2009). Available E-proteins (E2-2/TCF4, HEB/TCF12, and E2A/TCF3) can be bound by Musculin (MSC), which competes with MYOD1 for E-protein partners, and inhibits muscle gene activation (Yang et al., 2009). Additionally, one of the available E-proteins, E2A, in FN-RMS exists as a splice variant, termed E2A-2/5, which lacks exons 3 and 4, regions that encode the first activation domain. MYOD1:E2A-2/5 heterodimers can bind DNA, but are less efficient at transactivation than MYOD1:full length E2A heterodimers (Yang et al., 2009). Although in FN-RMS MYOD1 protein expression is high, MYOD1 binding is disrupted by sequestration of E-proteins by other TFs and overexpression of the E2A-2/5 splice variant, inhibiting optimal MYOD1 TF function.

MYOD1 is a master TF that participates with other TFs to cooperatively bind at large cis-regulatory enhancer regions which are critical for cell-type specification. Analysis of RNA-sequencing and H3K27ac chromatin immunoprecipitation sequencing (ChIPseq) data identified MYOD1, MYOG, SOX8, PAX7, and AP-1 family TFs as candidates for a FN-RMS specific CRC (Gryder et al., 2019a). The role of MYOG in a FN-RMS CRC is perplexing, as many proposed mechanisms for maintenance of FN-RMS tumors in a proliferative state involve inhibiting MYOD1’s ability to transactivate MYOG. Given some of the single-cell RNAseq data in FN-RMS tumors, and the inability of *MYOG* expressing cells to function as TPCs, it would be of interest to query the CRC specifically in TPCs (Wei et al., 2022; Danielli et al., 2023; Patel et al., 2022; Danielli et al., 2024). One TF for whom cooperative binding with MYOD1 at large enhancer regions has been demonstrated is SIX1 (Hsu et al., 2022). In two different FN-RMS cell lines it was found that SIX1 and MYOD1 cooperatively bind at enhancers associated with stem and proliferative states (Hsu et al., 2022). Cooperative binding between SIX1 and MYOD1 has also been demonstrated in models of mouse skeletal muscle development (Liu et al., 2010). A full characterization of TFs cooperatively occupying enhancers with MYOD1 in FN-RMS has yet to be completed and would provide critical insights into the transcriptional regulation of this deadly pediatric disease.

**FIGURE 3:**
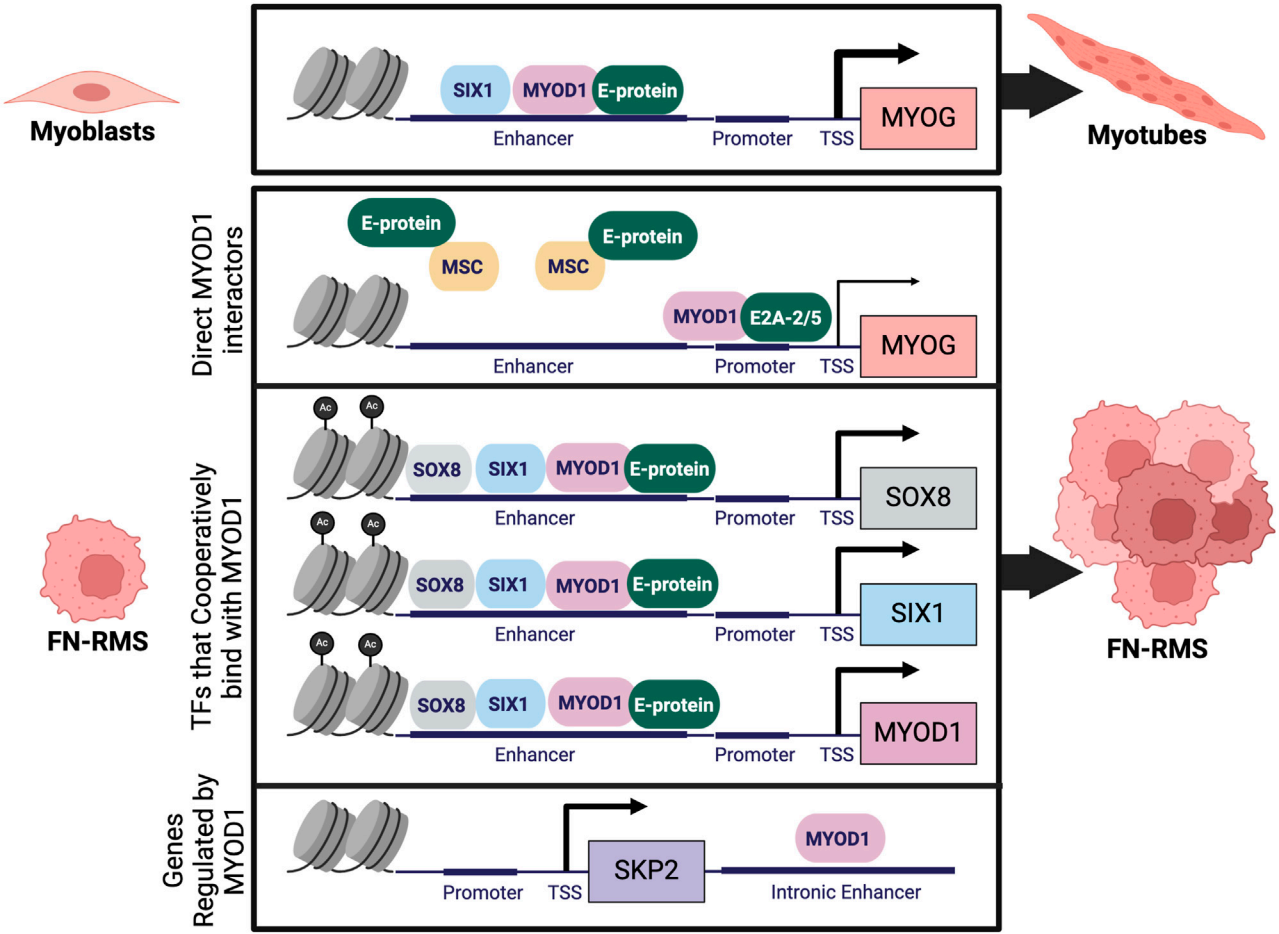
Dysregulation of MYOD1 in FN-RMS: During normal myogenesis MYOD1 forms a heterodimer with an E-protein to activate transcription of downstream genes like MYOG which regulate terminal skeletal muscle differentiation. In FN-RMS, MYOD1 is prevented from activating MYOG through dysregulation of direct MYOD1 interactors like E-proteins, and through formation of a CRC, resulting in cooperative binding of MYOD1 with other TFs. In the context of RMS, MYOD1 directly regulates transcription of novel targets like SKP2, enhancing cell proliferation. Figure created with Biorender.com.

Genes downstream of MYOD1 in FN-RMS are often critical for maintenance of oncogenic growth and proliferation pathways. ChIPseq performed for MYOD1 in primary human myoblasts and myotubes and a human FN-RMS cell line (RD) demonstrated key similarities as well as differences between MYOD1 binding in skeletal muscle cells and in FN-RMS (MacQuarrie et al., 2013). Many of the binding sites for MYOD1 are shared between myogenesis and FN-RMS, however, there are some sites with increased binding in FN-RMS, notably *CXCR4, SMOC1, GLI3,* and *ELMO1* (MacQuarrie et al., 2013). In addition, MYOD1 directly regulates *SKP2* (S-phase kinase associated protein-2) in RMS, a substrate recognition subunit of the E3 ubiquitin ligase complex that is necessary for tumor cells to maintain cell cycle progression (Pomella et al., 2023). Such binding thus inhibits differentiation while promoting continued proliferation. Furthermore, in RMS, MYOD1 is prevented from binding cis-regulatory regions associated with differentiation genes such as *MEF2C, RUNX1, JDP2*, and *NFIC* (MacQuarrie et al., 2013). Thus, in RMS, MYOD1 is prevented from binding E-boxes that enable differentiation, while maintained or redirected to stem and proliferation associated E-boxes to promote tumor survival and proliferation (MacQuarrie et al., 2013; Yang et al., 2009; Cao et al., 2010). Multiple mechanisms leading to dysregulated MYOD1 genomic binding and localization have been identified in FN-RMS and have been shown to promote oncogenic processes (Gryder et al., 2019a; Hsu et al., 2022; Tapscott et al., 1993; Yang et al., 2009; Pomella et al., 2023) (Figure 3).

The MRF *MYOG* (myogenin) is expressed during later stages of myogenesis, and in a majority of RMS (Zhang et al., 1995; Rekhi et al., 2018). Although *MYOG* is transcriptionally regulated by MYOD1 and expressed in RMS, the ability of *MYOG*+ cells to proliferate and reproduce the full FN-RMS tumor is debated (Yohe et al., 2018). In highly proliferative FN-RMS cells, *MYOG* is not commonly expressed (Wei et al., 2022; Patel et al., 2022). One school of thought is that MYOG expression is inhibited to prevent terminal differentiation of tumor cells. One mechanism through which *MYOG* expression is inhibited in FN-RMS is through the MAPK signaling pathway, whereby ERK2 binds the *MYOG* promoter and represses transcription (Yohe et al., 2018). Treatment of FN-RMS cell lines with trametinib, a MEK1/2 tyrosine kinase inhibitor, results in increased expression of differentiation TFs like *MYOG* and *MEF2C*, leading to terminal differentiation of tumor cells (Yohe et al., 2018). In transgenic FN-RMS zebrafish models, *myog+* tumor cells are less proliferative than *myf5+* cells, but are able to cross zebrafish myotomes, demonstrating a more migratory phenotype. Increased migration of *myog+* cells, compared to the stationary Myf5+ cells, resulted in segregation of the two cell populations, a phenomenon reproduced in human FN-RMS tumor samples (Ignatius et al., 2012). However, supporting the idea that suppression of *myog* is critical for RMS proliferation, *myog+* FN-RMS cells were largely non-proliferative (Ignatius et al., 2012). In summary, MYOG expression in FN-RMS causes cells to lose proliferative capacity, become more migratory, and gradually adopt a more differentiated state (Ignatius et al., 2012; Patel et al., 2022; Yohe et al., 2018).

Intriguingly, while there are limited genetic perturbations in FP-RMS, it appears that the PAX3/7-FOXO1 fusion protein alters the ability of MRFs to activate a myogenic program, thus facilitating tumor proliferation. PAX3-FOXO1 and - the much rarer - PAX7-FOXO1 translocation, when expressed in the murine mesenchymal progenitor cell line, C2C12, phenocopy dominant-negative PAX3 and PAX7 whereby they suppress myogenic differentiation and prevent expression of MYOD1 target genes like *Myog* and muscle creatine kinase (*Mck*) (Calhabeu et al., 2013). However, in this study, PAX3-FOXO1 or PAX7-FOXO1 fusion proteins did not inhibit MYOD1 from binding the *Myog* promoter, but rather decreased *Myog* transactivation (Calhabeu et al., 2013), by decreasing both RNA Polymerase II binding and histone H4 acetylation at the *Myog* promoter (Calhabeu et al., 2013). One limitation of this study is that previous work showed that expression of PAX3/7-FOXO1 alone is insufficient to induce tumor formation in most cell types, and requires the additional loss of a tumor suppressor ((Keller et al., 2004b; Curto et al., 2020)). This study demonstrates that PAX3/7-FOXO1 alters the ability of MYOD1 to activate downstream target genes.

**FIGURE 4:**
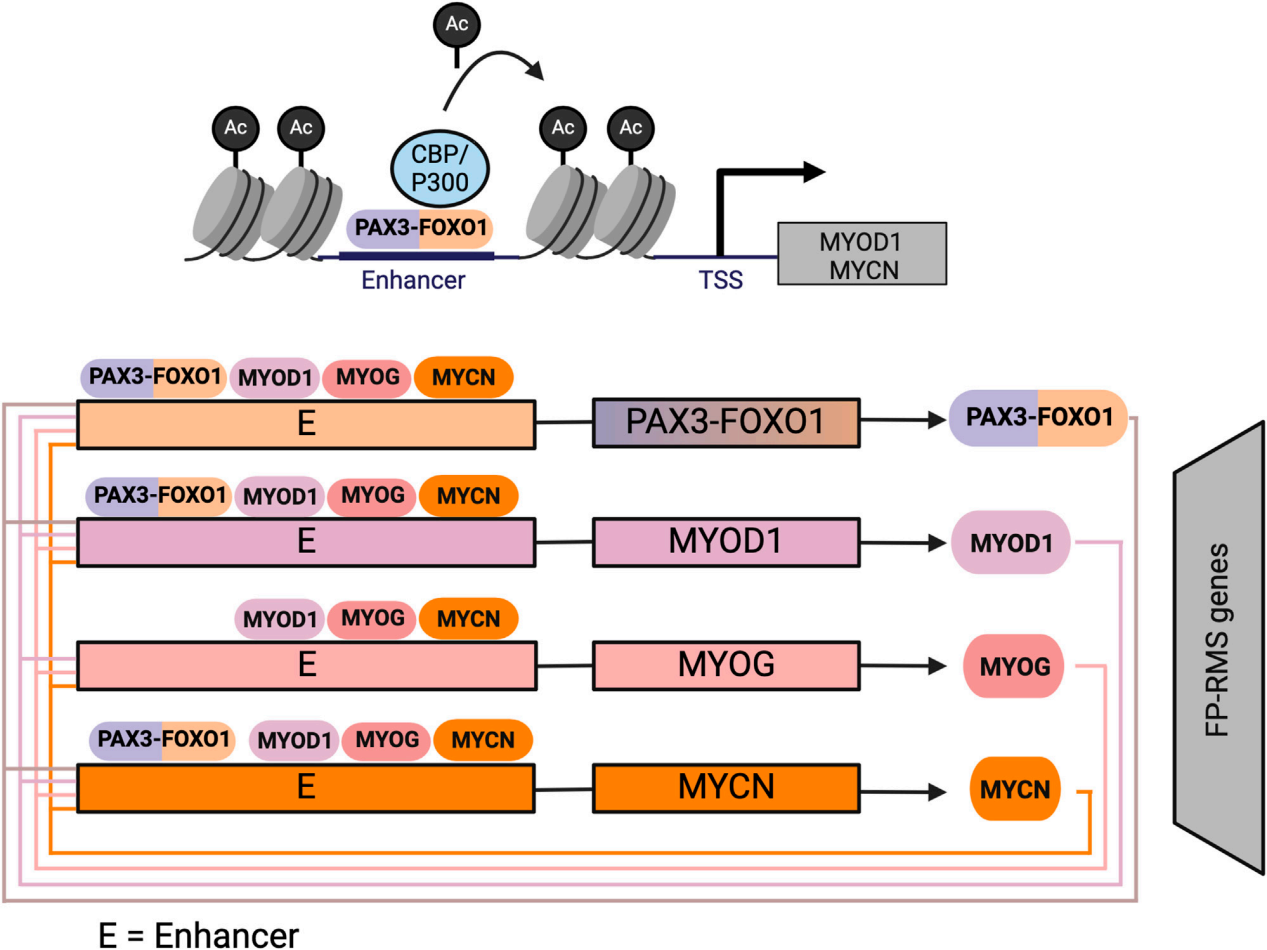
Core-regulatory circuit involving MYOD1 in FP-RMS: In FP-RMS PAX3-FOXO1 initiates expression of MYOD1, and MYCN, which form an autoregulatory circuit in FP-RMS whereby they bind at large enhancer regions to establish the FP-RMS transcriptome. Figure created with Biorender.com.

Critically, in FP-RMS *PAX3/7-FOXO1* is not the only genetic lesion, *MYCN* is frequently amplified. In human FP-RMS cell lines, PAX3-FOXO1 binds to the enhancers of key TFs, including *MYOD1* and *MYCN*, amplifying their expression (Figure 4) (Gryder et al., 2017). Both MYCN and MYOD1, in turn, bind to *MYOG* enhancers, along with MYOG itself, sustaining its expression (Gryder et al., 2017; Gryder et al., 2020). Binding of PAX3-FOXO1 to key TF genes results in a self-perpetuating gene regulatory loop: MYOD1, MYOG, and MYCN are required for PAX3-FOXO1 expression, as all master TFs bind to a FOXO1 super-enhancer that regulates the fusion protein’s expression (Gryder et al., 2020). This loop ensures the continuous expression of each core regulatory TF, maintaining tumor cells in a proliferative, myoblast-like state. MYOD1, MYOG, and MYCN, three master TFs, co-localize at highly active enhancer regions throughout the FP-RMS genome (Figure 4). In contrast, PAX3-FOXO1 occupies only half of highly active enhancer regions, which are defined as super-enhancers (Gryder et al., 2017). Cooperative binding between MYOD1, MYOG, MYCN, and PAX3-FOXO1 appears to hold FP-RMS cell lines in an undifferentiated state by maintaining activation of distal enhancers that are normally inactivated in later stages of myogenesis. For example, in mature skeletal muscle, the H3K27ac signal—a marker of active enhancers - decreases at loci such as MSC, MYOD1, MEST, and IGF2. However, in FP-RMS, the H3K27ac signal is maintained, in part through PAX3-FOXO1 gene occupancy (Gryder et al., 2017). These epigenetic alterations highlight how the PAX3-FOXO1 fusion protein sustains enhancer activation by partnering with key MRFs resulting in the unique regulatory landscape of FP-RMS. In FP-RMS cell lines, the presence of MYCN at the *MYOG* locus may explain the discordance between the finding that in C2C12 cells, PAX3-FOXO1 inhibits *Myog* gene activation by MYOD1, but in FP-RMS cell lines MYOD1 drives MYOG expression (Gryder et al., 2017). However, as in FN-RMS, scRNAseq data demonstrated variable *MYOG* expression in progenitor and differentiated RMS subpopulations (Patel et al., 2022; Danielli et al., 2024). Further investigating TF genomic localization in FP-RMS subpopulations may be important to further elucidate the respective contributions of PAX3/7-FOXO1 and MRFs to RMS proliferation.

### 2.4 Epithelial-mesenchymal transition (EMT) associated TFs: SNAI1, SNAI2, and TWIST2

Epithelial-mesenchymal transition (EMT) associated TFs, SNAI2 and TWIST2 compete with MYOD1 for binding at E-boxes in RMS, preventing MYOD1 from activating a myogenic differentiation program. As mentioned previously, in muscle development, SNAI1 and SNAI2 repressively bind at GC-rich E-boxes that are enriched in differentiation gene enhancers, preventing MYOD1 from binding. However, SNAI1 and SNAI2 do not compete with MYOD1 for binding at AT-rich E-boxes present in growth and proliferation enhancers (Soleimani et al., 2012). In later stages of myogenesis, *SNAI1* and *SNAI2* expression declines, allowing MYOD1 to re-localize to GC-rich E-boxes at differentiation genes (Soleimani et al., 2012). In FN-RMS, *SNAI2* is highly expressed, regulated by MYOD1, and competes with MYOD1 at E-box containing enhancers associated with genes that are necessary for terminal myogenic differentiation such as *MYOG*, *MEF2A/C/D*, and *CDKN1A* (Pomella et al., 2021). In human cell line models of FN-RMS, NOTCH1 regulates expression of *SNAI1*, increasing the number of proliferative, tumor propagating cells by repressing expression of *MEF2C* (Ignatius et al., 2017). This finding was extended to transgenic zebrafish models of FN-RMS where transgenic expression of NOTCH1 increased tumor incidence and the proportion of tumor propagating *myf5+* cells within the tumor (Ignatius et al., 2017). In studies of normal muscle development, premature myogenesis induced by expression of MYF5 and MYOD1 is inhibited in the presomitic mesoderm through activation of NOTCH1 signaling pathways (Kopan et al., 1994). Expression of NOTCH1 in FN-RMS may be a mechanism through which tumors inhibit execution of myogenic differentiation, a program co-opted from normal myogenesis. Implicating this mechanism in FP-RMS, *SNAI2* was identified as an early target of PAX3-FOXO1, opening the possibility that SNAI2 plays a role in regulating MYOD1 localization in FP-RMS tumors (Khan et al., 1999).

Another EMT-associated TF, TWIST2, represses myogenesis during normal development by competing for enhancer binding with MYOD1. However, in contrast to SNAI1, TWIST2 also inhibits MYOD1 through a direct interaction between the two proteins basic domains, and by sequestering E-proteins necessary for MYOD1 binding (Hamamori et al., 1997; Hebrok et al., 1997; Spicer et al., 1996). Amplification of *TWIST2* is observed in FN-RMS, and resulting increased levels of *TWIST2* redirect MYOD1 from myogenic loci to oncogenic loci (Hamamori et al., 1997; Hebrok et al., 1997; Li et al., 2019). While some of the pro-oncogenic effects of TWIST2 and SNAI2 are attributed to direct competition with MYOD1 for E-box binding, some changes in expression mediated by these TFs are also due to more global alterations in chromatin structure (Soleimani et al., 2012; Li et al., 2019). For example, at loci where TWIST2 competes with MYOD1 for binding, a significant decrease in H3K27ac deposition was observed concomitant with an increase in H3K27me3 (a repressive mark) (Li et al., 2019). This data demonstrates that TWIST2 not only blocks MYOD1 from activating expression of myogenic differentiation genes but also represses them. EMT-associated TFs prevent MYOD1 from binding differentiation loci in FN-RMS by both occupying the E-boxes at which MYOD1 would normally bind and by increasing repressive chromatin marks at this locus, resulting in maintenance of FN-RMS cells in a less differentiated, and more proliferative state.

### 2.5 YAP/TAZ, TEAD transcription factors and the hippo signaling pathway

Transcriptional regulators associated with adult skeletal muscle are also implicated in RMS tumorigenesis. Upstream signaling through the hippo pathway causes repression of the transcriptional coactivators YAP and TAZ, by phosphorylation of YAP Ser127 and TAZ Ser89, leading to sequestration of these proteins in the cytoplasm (Zhao et al., 2007; Mohamed et al., 2016). When hippo signaling is inactive, YAP and TAZ can translocate to the nucleus where they bind TEAD1-4 TFs to activate genes associated with organ growth and regeneration (Driskill and Pan, 2023). *YAP1* expression is upregulated in FN-RMS and its localization to the nucleus has been identified as one of the initial genetic events necessary to induce FN-RMS formation (Slemmons et al., 2015; Tremblay et al., 2014). Interestingly, in FN-RMS cell lines, KD of *YAP1* results in increased expression of MRFs, *MYOD1*, *MYOG*, and *MRF4* (Slemmons et al., 2015). In two FN-RMS cell lines, KD of *YAP1* resulted in increased myogenic differentiation (Slemmons et al., 2015). These data indicate that in FN-RMS, inactive hippo signaling and resultant YAP1 nuclear localization led to suppression of myogenic differentiation. Indeed, constitutive YAP1 nuclear localization in non-quiescent satellite cells in a GEMM resulted in FN-RMS formation (Tremblay et al., 2014). In this context, YAP1 and TEAD1 interact to activate expression of genes associated with proliferation and oncogenesis and repress genes associated with differentiation (Tremblay et al., 2014).

Expression of TAZ, a paralog of YAP1, is associated with worsened survival in FN-RMS patients, and 12% of FN-RMS patients have copy number gains at the *WWTR1* (*TAZ*) chromosomal locus (Mohamed et al., 2016). While KD of *YAP1* and *WWTR1* in FN-RMS cell lines both result in decreased cell proliferation, there are distinct differences in how these two genes influence differentiation (Mohamed et al., 2016; Slemmons et al., 2015). KD of *YAP1* increases expression of myosin heavy chain (*MyHC)* and expression of MRFs associated with terminal differentiation (Slemmons et al., 2015). In contrast*, WWTR1* KD does not result in an increase in MyHC expression, indicating these two paralogs are transcriptionally co-activating different genes in FN-RMS (Mohamed et al., 2016). These data recapitulate those seen in normal development, where both YAP and TAZ, when expressed in satellite cells, promote proliferation. However, in later stages of myogenesis, TAZ promotes myogenic differentiation while YAP inhibits it (Sun et al., 2017). The different pathways regulated by YAP and TAZ in myogenesis and FN-RMS may be a result of differential regulation of TEAD TFs. One challenge in studying TEAD TFs is that they have overlapping expression and functional redundancy. For example, KD of *Tead1*, *Tead2*, or *Tead4* in primary myoblasts does not alter myotube formation. However, combinatorial KD of *Tead1* and *Tead4* or *Tead1*, *Tead2*, and *Tead4* does significantly shorten myotube length and decrease the number of muscle cells that initiate expression of *MyHC* (Joshi et al., 2017). Critical to the hypothesis that differential regulation of TEAD TFs by YAP or TAZ regulates myogenic differentiation, there is evidence that TEAD1 and TEAD4 contribute to myogenesis through non-redundant functions. ChIPseq for TEAD1 and TEAD4 in differentiating C2C12 cells demonstrates that during differentiation there is a switch from TEAD1 and TEAD4 genomic occupancy in myoblasts, to only TEAD4 occupancy in differentiated cells (Joshi et al., 2017). TEAD1 and TEAD4 co-occupied genes are associated with TGF-β, WNT, and Hippo signaling pathways in undifferentiated C2C12 cells, and in differentiated cells, TEAD4 only occupied loci were annotated to genes associated with skeletal muscle architecture, and differentiation (Joshi et al., 2017).

Similar to what is observed in FN-RMS, the Hippo signaling pathway facilitates tumorigenesis in FP-RMS. Studies have shown that in FP-RMS cells, TAZ is localized to the nucleus to a greater extent when compared to mouse myoblast cells, indicating that the repressive Hippo signaling pathway is less active, allowing dephosphorylated TAZ to localize to the nucleus and function as a coactivator with TEAD TFs (Deel et al., 2018). Interestingly, in FP-RMS, YAP1 is primarily localized to the cytoplasm, indicating that the hippo signaling pathway may differentially regulate the two effector co-activators in the same cell (Tremblay et al., 2014). When FP-RMS cell lines are serially passaged as rhabdospheres, *TAZ* mRNA expression increases, indicating that this signaling pathway may be best studied in 3D cell-culture models. Functionally, FP-RMS 3D-cultured cell-line models demonstrate increased expression of stem cell markers, *SOX2, NANOG,* and *OCT4,* and KD of *WWTR1* (TAZ) in 3D-cultured cells resulted in reduced sphere forming frequency (Deel et al., 2018). Genetic experiments demonstrate that *WWTR1* KD inhibits FP-RMS growth in both *in vitro* and in *in vivo* models of disease (Deel et al., 2018). These studies demonstrate that TAZ activity is necessary for maintaining stem cell markers expression and proliferation in FP-RMS cell lines (Deel et al., 2018). The mechanism through which the hippo signaling pathway and its effectors, YAP/TAZ and TEAD TFs, regulate growth and proliferation in FP-RMS remains to be uncovered. Gaining a better understanding of the respective contributions of different TEAD TFs to myogenesis and their relative importance to RMS pathogenesis is critical to exploring new targeted therapeutic avenues. Presently there are a variety of allosteric and direct inhibitors of the YAP/TAZ-TEAD interaction, as well as other agents targeting TEAD TFs in development (Chapeau et al., 2024; Hagenbeek et al., 2023). Understanding the specifics of how YAP and TAZ work with TEAD TFs to regulate RMS cell proliferation will be critical to optimizing the therapeutic use of compounds targeting this pathway.

### 2.6 GLI transcription factors and the hedgehog signaling pathway

The incidence of FN-RMS is elevated, though still rare, in Gorlin Syndrome (nevoid basal cell carcinoma syndrome) patients, a congenital disorder where *PTCH1* is mutated (Teglund and Toftgård, 2010; Hettmer et al., 2015). This finding strongly implicates the Hedgehog (Hh) signaling pathway in RMS pathogenesis. In mouse models and genetic studies, constitutive activation of the Hh signaling mediates FN-RMS formation. This pathway is controlled upstream by Patched (PTCH1), which inhibits the Smoothened (SMO) G-coupled protein receptor, enabling the proteolytic cleavage of the full-length GLI TF into repressive GLI (GLIR). Translocation of GLI to the nucleus results in repression of GLI target genes (Skoda et al., 2018; Pak and Segal, 2016). In the presence of a Hh ligand, PTCH1 is degraded, releasing repression of SMO, which then promotes suppressor of fused (SUFU) and GLI dissociation, allowing activated GLI (GLIA) to move to the nucleus and promote target gene transcription (Skoda et al., 2018; Pak and Segal, 2016). There are three GLI family members: GLI1 is a transcriptional activator, GLI2 is primarily a transcriptional activator, and GLI3 is a transcriptional repressor (Pak and Segal, 2016). In RMS patients, high *PTCH1* expression is correlated with reduced overall survival (Zibat et al., 2010). While this result may suggest that Hh signaling inhibits RMS, *PTCH1* is a GLI transcriptional target gene, and thus expression of this transcript indicates Hh signaling pathway activation (Skoda et al., 2018). In another study, 50% of FN-RMS patients had low-level gains (log_2_ > 0.2) in the genomic region containing *GLI1* (Paulson et al., 2011). Interestingly, GLI1 and one of GLI1’s transcriptional targets, the ATP-binding cassette sub-family B member 1 (*MDR1*) are upregulated in vincristine-resistant RMS cell lines, offering a potential explanation for compound resistance (Yoon et al., 2020). Taken together, these data strongly suggest that Hh signaling is activated in FN-RMS and plays a role in severity of the disease and mechanisms of chemoresistance.

Several genetic studies modeling RMS have demonstrated the importance of Hh signaling activation on RMS tumor formation. In GEMMs, mice with global heterozygous *Ptch1* KO, or tamoxifen inducible global expression of *Smo* ^*M2*^, a constitutively active smoothened, develop FN-RMS (Hahn et al., 2000; Lee et al., 2007; Mao et al., 2006). Interestingly, mice with a conditional *Smo* ^*M2*^ allele that is expressed in adipose-protein 2 (aP2) expressing cells develop tumors resembling FN-RMS with a higher penetrance (80%) than mice ubiquitously expressing *Smo* ^*M2*^ (Hatley et al., 2012). Follow-up fate-mapping experiments revealed that endothelial cells were the *Smo* ^*M2*^ expressing cells of origin for FN-RMS in mice (Drummond et al., 2018). This finding is especially notable given that studies in muscle development have shown that Hh signaling is important in the maintenance, but not initiation, of Myf5 expression (Chiang et al., 1996). It is possible that Hh signaling is a mechanism through which FN-RMS tumors maintain early MRF expression, resulting in sustained growth and proliferation. The role of constitutively active Hh signaling in the formation of RMS has been clearly demonstrated through genetically engineered mouse models of disease, and the importance of this signaling pathway and downstream GLI TFs have been demonstrated in human FN-RMS.

## 3 Therapeutic opportunities

Current therapeutic regimens rely on non-specific tumor treatment modalities, including chemotherapy, radiation, and surgery, which result in significant morbidity for patients with RMS. Targeting developmental TFs, aberrantly re-expressed in the context of RMS – several which are “oncofetal genes”- is a promising method to specifically target malignant tissue, while limiting toxicity to non-malignant tissues that typically do not express these TFs. Because of the interconnected nature of myogenic TFs and the genes they regulate, targeting a specific core regulatory TF, like MYOD1, or PAX3/7-FOXO1 is likely to collapse the tumor transcriptome resulting in terminal differentiation of tumor cells or tumor cell death. Currently, drugs that target epigenetic factors, BAF complex members, and histone deacetylases (HDACs), have shown therapeutic efficacy by abolishing large regulatory enhancer regions necessary for RMS tumor survival (Laubscher et al., 2021; Gryder et al., 2019b). While potentially effective, a significant concern for these drugs is the possibility of limited clinical efficacy due to a narrow therapeutic window arising from effects on non-malignant cells (DiNardo et al., 2023).

TFs have been considered “undruggable” as their protein structures generally lack highly structured regions like enzymatic binding pockets. One emerging therapeutic strategy is targeting TFs for selective degradation using Proteolysis Targeting Chimeras (PROTACs) and molecular glues. PROTACs consist of two synthetic ligands, one of which binds to E3 ubiquitin ligase and the other binds to the protein-of-interest (POI), connected by a linker. The PROTAC then functions as a tether, connecting the POI to an E3 ubiquitin ligase, resulting in POI polyubiquitination and proteasomal degradation (He et al., 2022). A strength to PROTACs is that they may be developed in a modular fashion as many ligands that bind E3 ubiquitin ligases are known. Therefore, once a suitable ligand is identified for the POI, first stage PROTAC development can be undertaken by linking the two ligands (He et al., 2022). In contrast, molecular glues are monovalent small molecules that simultaneously interact with the POI’s surface and the surface of E3 ubiquitin ligase (Sasso et al., 2022), also resulting in the degradation of the POI. Molecular glue chemical discovery is challenged by their structure – as they are monovalent- thus rational design to develop interactions with unstructured regions of TFs or other proteins can be challenging (Sasso et al., 2022). Despite these challenges, immense success has been realized in the field of molecular glues. Well known therapeutics like thalidomide, lenalidomide, and pomalidomide are molecular glues approved for the treatment of hematologic malignancies, and their mechanism of action has been shown to be via targeting C2H2 zinc finger containing TFs, such as IKZF1 and IKZF3, for selective degradation (Sievers et al., 2018). Thus, TF degradation mediated by E3 ubiquitin ligase is already being leveraged clinically for treatment of cancers reliant on IKZF1 and IKZF3, suggesting a powerful new therapeutic opportunity for potential development in the treatment of RMS. Additional approaches to selectively degrade TFs remains an area of significant interest for cancer therapeutics. Other approaches, for example, novel constructs using a dsDNA oligonucleotide containing a TF motif linked to an E3 receptor binding molecule may result in selective degradation of TFs without a TF binding ligand (Samarasinghe et al., 2021; Li et al., 2023). The stability, specificity and dosing of these so called “TRAFTACs” remains unknown, however these are an area of active exploration and useful tool compounds to explore the biological consequences of TF loss. Thus, developing an understanding of the role of essential TFs in normal myogenesis and in RMS is critical for future potential tumor-specific TF targeting as a means to inhibit RMS progression.

## 4 Conclusion

RMS is a disease characterized by the myogenic cell-identity that tumor cells acquire. This identity is maintained by constitutive expression of developmental TFs, which in normal myogenesis are carefully regulated by intracellular and extracellular cues. In RMS these developmental TFs become dysregulated, allowing for aberrant expression and genomic localization, resulting in an altered epigenetic landscape characteristic of RMS. While some RMS are driven by mutations in pathways common across cancer subtypes, sustained developmental TF expression is an intriguing attribute of these tumors. As discussed herein, TFs that regulate the myogenic identity in RMS are tumor dependencies, whose expression regulate the transcriptome of RMS to allow for sustained proliferation and evasion of differentiation. Increasing our understanding of gene regulatory networks in the context of embryonic myogenesis and applying those findings to RMS has provided powerful insights into a pediatric disease with a high degree of morbidity and mortality, and has revealed novel, potentially tumor specific, therapeutic targets.
